# Predictive Modeling of Shear Strength of Enzyme-Induced Calcium Carbonate Precipitation (EICP)-Solidified Rubber–Clay Mixtures Using Machine Learning Algorithms

**DOI:** 10.3390/polym17070976

**Published:** 2025-04-03

**Authors:** Qiang Ma, Meng Li, Hang Shu, Lei Xi

**Affiliations:** 1 Hubei Key Laboratory of Environmental Geotechnology and Ecological Remediation for Lake & River, Hubei University of Technology, Wuhan 430068, China; maqiang927@163.com (Q.M.); lmeng1014@163.com (M.L.); leixi@whut.edu.cn (L.X.); 2 Key Laboratory of Intelligent Health Perception and Ecological Restoration of Rivers and Lakes, Ministry of Education, Hubei University of Technology, Wuhan 430068, China; 3 Hubei Provincial Ecological Road Engineering Technology Research Center, Hubei University of Technology, Wuhan 430068, China

**Keywords:** shear strength, soil solidification, enzyme-induced carbonate precipitation, rubber, rubber–clay mixtures, machine learning, predictive modeling

## Abstract

The development of reliable predictive models for soil behavior represents a crucial advancement in geotechnical engineering, particularly for optimizing material compositions and reducing experimental uncertainties. Traditional experimental approaches for determining the optimal rubber particle size and content are often resource-intensive, time-consuming, and subject to significant variability. In this study, the shear strength of clay mixed with rubber particles solidified by the Enzyme-Induced Calcium Carbonate Precipitation (EICP) technique was investigated and predictively modeled using a machine learning algorithm. The effects of different rubber contents and particle sizes on the shear strength of the clay were analyzed experimentally, and a hybrid model of a convolutional neural network (CNN) and long short-term memory (LSTM) network optimized based on the crown porcupine optimization (CPO) algorithm was proposed to predict the shear strength of the EICP-treated clay mixed with rubber particles. The superiority of the CPO-CNN-LSTM model in predicting shear strength was verified by comparing multiple machine learning algorithms. The results show that the addition of rubber particles significantly improves the shear strength of the clay, especially at a 5% rubber content. The coefficient of determination (*R*^2^) of the CPO-CNN-LSTM model on the training and test datasets reaches 0.98 and 0.97, respectively, which exhibit high prediction accuracy and generalization ability.

## 1. Introduction

The global focus on achieving ‘low carbon’ objectives and ‘carbon neutrality’ has driven the exploration of sustainable techniques for soil enhancement. In recent years, a significant body of research has explored various eco-friendly technologies, with biocementation through urea hydrolysis emerging as a promising approach for soil enhancement that reduces CO_2_ emissions [1,2]. Compared to traditional chemical treatments, biocementation is more environmentally sustainable and poses fewer risks to human health. Specifically, techniques such as Microbial-Induced Carbonate Precipitation (MICP) and Enzyme-Induced Carbonate Precipitation (EICP) have garnered considerable attention for their potential to improve soil properties [3]. MICP relies on urease produced by microorganisms, whereas EICP directly utilizes extracted urease to catalyze the necessary reactions, thereby eliminating the need for associated bacteria [4]. Furthermore, the dimensions of the isolated urease, averaging 0.012 μm, are notably smaller compared to those of microbially synthesized enzymes, which generally measure between 0.3 and 0.5 μm. This size advantage allows EICP to be more adaptable to a wider range of soils and simplifies application procedures. Moreover, potential competition between native soil microorganisms and bacteria introduced via the MICP technique [5] underscores the superior advantages of EICP. Consequently, the EICP approach exhibits greater environmental compatibility and operational efficiency compared to MICP, rendering it applicable to a wider range of soil types and featuring a more simplified implementation process [6,7]. Recent research has confirmed that EICP effectively improves the strength and rigidity of sandy soils [8,9,10,11]. However, most investigations have predominantly concentrated on sandy soils, with limited attention given to fine-grained soils. In cohesive soils, prevalent engineering issues include inadequate strength, excessive compressibility [12], substantial settlement [13], and vulnerability to swelling and shrinkage caused by seasonal changes [14,15]. Therefore, research on the application of EICP in clayey soils is critically important.

In addition to the advantages of EICP itself, the application of some auxiliary materials in research on soil improvement has also received attention, such as rubber particles in soil improvement, which is of great importance. Incorporating rubber particles into clay to produce lightweight backfill materials not only reduces the self-weight stress in filling projects but also mitigates the environmental pollution caused by waste tires [16,17]. Studies have demonstrated the versatility of waste tires in civil engineering applications, including their use in soil stabilization [18], road construction materials [19], vibration damping systems [20], and slope reinforcement [21]. The incorporation of rubber as an eco-friendly material not only enhances soil characteristics but also significantly lowers reinforcement expenses [22,23,24,25,26,27,28]. Cui et al. [29] investigated the incorporation of rubber particles into biocemented calcareous sands, which was shown to mitigate brittleness through delayed dilation mechanisms, thereby improving overall mechanical performance. Chen et al. [30] systematically evaluated EICP-treated loess reinforced with rubber additives, revealing significant enhancements in shear strength parameters (cohesion and internal friction angle). Their experimental results further established optimal reinforcement conditions utilizing rubber particles within the 1–2 mm size range at a 10% mass fraction.

Shear strength is a critical parameter for evaluating the mechanical behavior of soils. Traditional methods for determining shear strength, such as triaxial compression testing, are not only time-consuming and costly but also highly dependent on the performance of equipment and the skill of operators. In contrast, machine learning algorithms have demonstrated significant potential in accurately and efficiently predicting soil properties [31,32,33,34,35]. The implementation of machine learning methods in the study of new materials and their modifications has seen significant progress in recent years. Many studies have demonstrated the efficacy of machine learning algorithms in predicting the mechanical properties of composites and the potential for optimizing material composition to improve performance [36,37,38,39,40,41,42,43]. These algorithms excel at handling complex, nonlinear relationships between input variables and output responses, making them particularly well suited for predicting the shear strength of EICP-treated soils. However, the application of advanced machine learning models, such as convolutional neural networks (CNNs) and long short-term memory (LSTM) networks, in geotechnical engineering remains in its early stages, especially concerning the prediction of biocemented soil behavior.

Therefore, this study aims to develop a novel machine learning model, CPO-CNN-LSTM, for predicting the shear strength of EICP-treated rubber–clay mixtures. This model combines the capabilities of convolutional neural networks (CNNs) and long short-term memory (LSTM) networks, enhanced by the Crested Porcupine Optimizer (CPO) algorithm, to efficiently identify spatial and temporal patterns within data. This study has three main goals: (1) to examine how rubber content and particle size affect the shear strength of EICP-treated clay; (2) to design and validate the CPO-CNN-LSTM model for precise shear strength prediction; and (3) to evaluate the performance of the proposed model against other widely used machine learning methods. By accomplishing these goals, this research aims to deliver a dependable tool for forecasting the mechanical properties of EICP-treated soils, promoting their wider use in geotechnical engineering applications.

## 2. Materials and Methods

### 2.1. Test Material

**Figure 1:**
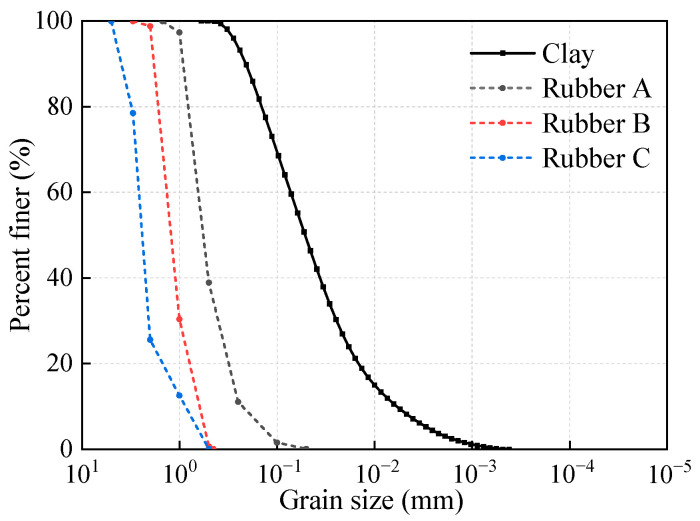
Particle size distribution of the clay and rubber particles.

**Figure 2:**
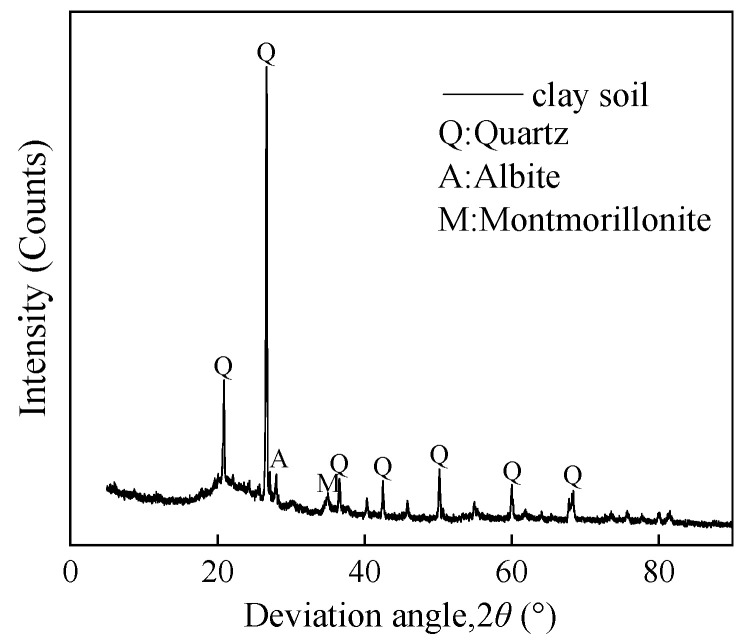
XRD diagram of the clay.

The soil used in this study was collected from a project excavation site in Hubei, China. Following a drying procedure, the basic physical parameters of the soil matrix were documented, as shown in Table 1. The particle size distribution curve of the soil used in the test is shown in Figure 1. Table 2 shows the composition of the soil tested by X-ray fluorescence spectroscopy (XRF). XRD analyses showed that the soil was dominated by quartz, sodium feldspar, and montmorillonite clay minerals, and the results of the tests are shown in Figure 2.

**Figure 3:**
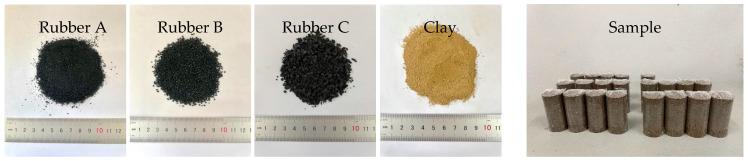
Different sizes of crumb rubber and clay and samples used in this study.

The rubber particles used in this study were obtained from waste tires, and the particle size distribution and engineering properties of the rubber particles are summarized in Figure 1 and Table 3. As shown in Figure 3, the particle size distributions of Rubber A, Rubber B, and Rubber C were similar to those of fine (0.075–0.425 mm), medium (0.425–2 mm), and coarse (2–4.75 mm) sand, respectively. The coefficient of inhomogeneity (*C*_u_) and coefficient of curvature (*C*_c_) were 2.95 and 1.12 for Rubber A. On the contrary, the *C*_u_ values for Rubber B and Rubber C were 2.20 and 2.94, respectively, and the corresponding *C*_c_ values were 1.05 and 1.81, respectively.

The EICP solution was prepared by mixing equal volumes of the EICP cementing solution and urease solution. The EICP cementing solution was formulated by combining urea and calcium acetate in a 1:1 molar ratio. Skimmed milk powder was then added to provide nucleation sites [44]. Considering the inhibitory effects of high-concentration salt solutions on urease activity and their influence on the physical properties of cohesive soil [45], a lower concentration of the EICP cementing solution (0.5 M urea, 0.5 M calcium acetate, 0.684 g/L urease, and 4 g/L skimmed milk powder) was used to enhance the effectiveness of EICP. The activity in the urease solution was measured using the electrical conductivity (EC) method [46].

### 2.2. Specimen Preparation

The clay collected from the site was oven-dried and screened through a 2 mm sieve. Subsequently, rubber particles were incorporated into the clay at 2.5%, 5%, 7.5%, and 10% by dry weight of soil under dry conditions, followed by thorough mixing. The modified Proctor compaction test was employed to determine the maximum dry density and optimum water content for the various clay–rubber particle combinations. The EICP solution was then added to the mixture and blended according to the optimum moisture content. The mixture was pressed into a mold using a hydraulic jack and allowed to stand for one minute. After molding, the samples were removed, sealed with plastic film, and placed in a curing box maintained at 20 °C and 95% relative humidity for 14 days.

### 2.3. Triaxial Compressive Test

The TSZ-2 strain-controlled triaxial instrument (Nanjing Huade Soil Instrument Manufacturing Co., Ltd., Nanjing, China) was used to perform consolidated–drained triaxial compression tests. A 39.1 mm × 80 mm triaxial sample was used, and the tests were conducted at a loading rate of 0.4 mm/min [47]. The confining pressures were set at 50 kPa, 100 kPa, and 150 kPa. Before the test, each specimen was fully saturated through a three-step method: (1) vacuum saturation for 24 h before testing; (2) hydraulic saturation for 30 min after installation on the apparatus; and (3) counterpressure saturation until at least 95% saturation was achieved. The specimens were then consolidated under a specific confining pressure. After consolidation, axial compression was performed at the specified rate until an axial strain of 20% was reached.

### 2.4. Crested Porcupine Optimizer (CPO)

In this study, a novel nature-inspired optimization algorithm, the Crested Porcupine Optimizer (CPO) (MATLAB R2022a v9.12) [48], is used, which is inspired by the defense behavior of crown porcupines in nature. The algorithm can effectively solve complex optimization problems by simulating the four defense mechanisms of the crown porcupine (sight, sound, smell, and physical attack) and shows especially high performance in large-scale problems. The first two defense mechanisms (vision and sound) reflect the global exploration behavior of the CPO, while the last two (odor and physical attack) correspond to local exploitation behavior. The CPO achieves a dynamic balance between the exploration and exploitation phases of the optimization process by integrating the synergistic effects of these four defense strategies. In addition, the CPO algorithm introduces an innovative cyclic population reduction technique, i.e., reducing the population size N in each generation, which simulates the reality that not all crested porcupines activate their defense mechanisms when faced with a threat; rather, only the threatened individuals do. This strategy not only speeds up the convergence of the algorithm but also enhances the diversity of the population, thus improving the optimization ability of the algorithm and the likelihood of avoiding falling into local optima. Similarly to other algorithms based on meta-heuristic populations, the CPO starts by performing a search based on an initial set of individuals (candidate solutions), each of which assumes some kind of defense and is evaluated by a fitness function (or objective function).

### 2.5. Convolutional Neural Network (CNN)

**Figure 4:**
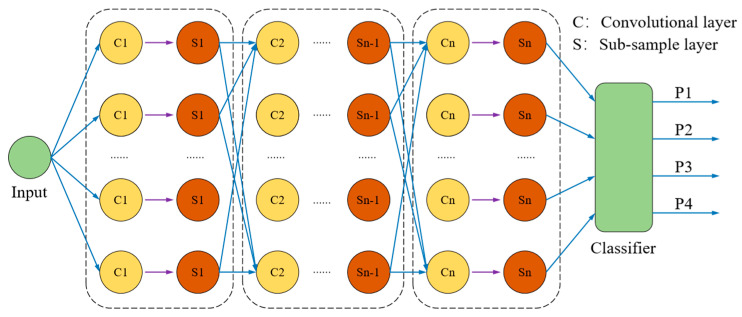
Deep hierarchy of CNN models.

The research adopts a convolutional neural network (CNN) [49], a deep learning paradigm that realizes multi-dimensional data feature learning through the layer-by-layer abstraction mechanism of the convolutional kernel and shows breakthroughs in the fields of image recognition, speech processing, natural language understanding, etc. The core architecture of the CNN consists of an input layer, a stacked convolutional layer, a pooling layer, and a fully connected layer, which form a systematic feature processing link. The convolutional layer extracts the spatial topological features of the input data through the mechanism of local perceptual field and weight sharing; the pooling layer adopts a downsampling strategy to compress the feature dimensions, which can reduce the number of parameters in the model and the computational complexity while retaining key information; and the fully connected layer establishes higher-order nonlinear associations through feature spatial mapping to finally complete the classification and prediction tasks. In this study, a CNN-based feature learning framework is constructed, and its hierarchical feature extraction mechanism effectively identifies the key factors of shear strength and reveals the high-order nonlinear correlations implied by the data. The deep-level hierarchical structure of the CNN model is shown in Figure 4.

### 2.6. Long Short-Term Memory (LSTM) Network

**Figure 5:**
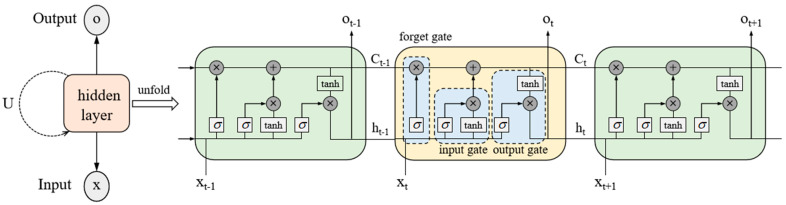
Typical structure of LSTM.

Long short-term memory (LSTM) [50], as a special kind of Recurrent Neural Network (RNN), has become a widely used benchmark algorithm in the field by virtue of its significant advantage in effectively handling long-term dependencies in time series modeling. As shown in Figure 5, the standard LSTM network structure and its unfolded form contain four core components: three gating mechanisms and a memory cell state. Among these, the memory cell state, as the core element of the LSTM architecture, assumes the function of an information transmission channel. The mechanism is regulated by three independent gating units (forget gate, input gate, and output gate) to realize the dynamic updating and maintenance of cell state information, so as to achieve the precise addition or deletion operation of information during the processing of the whole sequence.

### 2.7. CPO-Optimized CNN-LSTM Models

**Figure 6:**
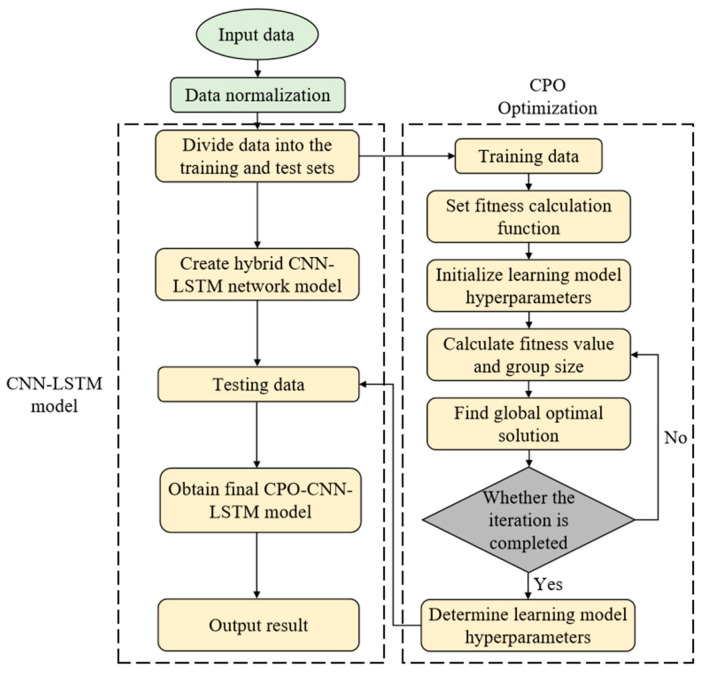
CPO-CNN-LSTM neural network prediction model.

In the parameter optimization process of CNN-LSTM models, hyperparameter configuration has a decisive impact on the model’s prediction performance. This study proposes an adaptive selection framework for hyperparameters based on the CPO algorithm, which focuses on optimizing key parameters such as the number of nodes in the hidden layer, the initial learning rate, and the L2 regularization coefficient. The algorithm execution process consists of six core stages: (1) constructing the fitness evaluation function; (2) initializing the hyperparameter search space of the model; (3) calculating the population fitness and determining the optimal size; (4) executing the search for the global optimal solution; (5) setting up an iterative termination-condition determination mechanism and returning to step (3) to continue searching for the optimal solution when the condition is not satisfied; and (6) outputting the optimized hyperparameter combinations. The system architecture and algorithm implementation flow of the CPO-CNN-LSTM prediction model constructed based on this optimization framework are shown in Figure 6. The integrated method significantly improves the model’s ability to capture complex timing features and generalization performance through multi-objective collaborative optimization.

### 2.8. CPO-CNN-LSTM Model Training

**Figure 7:**
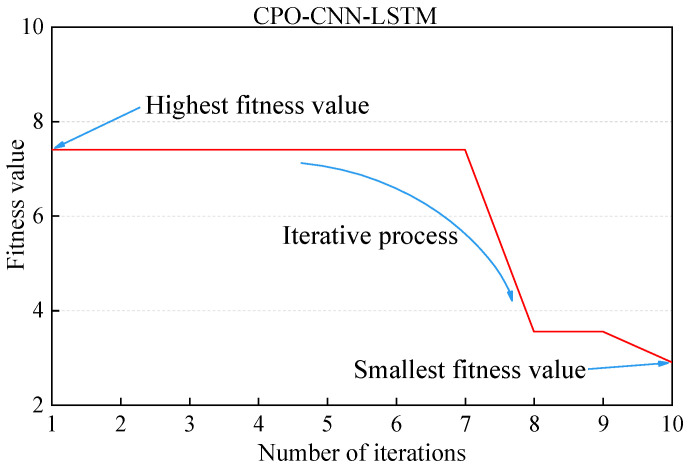
CPO-CNN-LSTM adaptation curve.

In this study, a CPO-CNN-LSTM method is used, which ingests data and learns from them in order to achieve accurate predictions of shear strength. The machine learning algorithm aims to construct the best prediction model by finding the optimal combination of hyperparameters. To this end, the algorithm initially sets the hyperparameters to random values and optimizes these hyperparameters using a cost function. Through a learning process on the training set, the algorithm ultimately determines the hyperparameter configurations that produce the best predictions. In order to evaluate the prediction performance of the model, this study randomly divides the dataset into two parts: the training set and the test set. In this case, the training set contains 80% of the total data, while the remaining 20% is assigned to the test set. This dataset division strategy is a routine practice in the field of machine learning to test the generalization ability of a model on an independent dataset, thus verifying the validity and reliability of the model. The dataset contains eight input variables and one output variable, totaling 117 datasets. The input variables include enzyme activity, cement concentration, percentage of clay, percentage of rubber particles, average particle size of rubber particles, coefficient of inhomogeneity, coefficient of curvature, and confining pressure. The output variable is the shear strength of the EICP-solidified rubber–clay mixtures. The machine learning model optimized by the CPO algorithm exhibits significant performance improvement. As shown in Figure 7, in the 8th iteration, the fitness value was significantly reduced to about 3.5, which signifies that the algorithm has converged to a more optimal solution, further confirming its effectiveness in the parameter optimization of the shear strength prediction model.

## 3. Results and Discussion

### 3.1. Shear Strength

Shear strength is a key index for evaluating the performance of soils and represents the ability of soils to resist damage under shear stress. Traditional laboratory studies determine the shear strength of soil samples through triaxial compressive tests. In this study, the effects of different rubber particle sizes, contents, and confining pressures on the shear strength of EICP-cured rubber–clay mixtures were investigated through triaxial compression tests.

**Figure 8:**
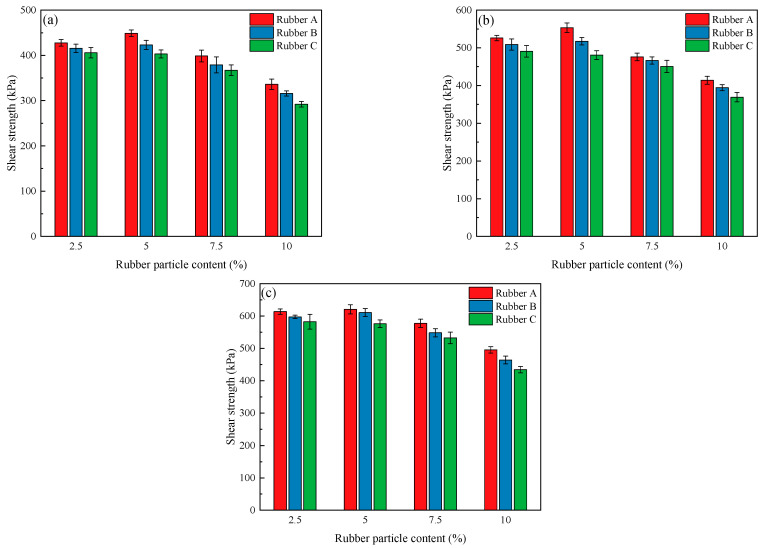
Shear strength variation with rubber particle size and content under different confined pressures: (**a**) 50 kPa, (**b**) 100 kPa, and (**c**) 150 kPa.

Figure 8 depicts the variation in the shear strength of EICP-treated clay mixed with rubber particles with different rubber particle sizes and contents at different confined pressures. It can be seen in the figure that shear strength increases and then decreases with the increase in rubber particle doping. The highest shear strength was observed when the rubber particle content was 5%. This is consistent with other findings [51,52] indicating that the incorporation of rubber particles increases strength and suggests that the elastic response of rubber particles during compression helps to prevent cracking [53]. In addition, EICP has a reinforcing effect between rubber particles and clay particles [54]. For the same content of rubber particles, shear strength tends to decrease with the increase in the size of rubber particles. This phenomenon can be attributed to the fact that the incorporation of small-sized rubber powder increases the area of the relatively flat surface of the soil, reduces the pore volume, and smooths the surface [30]. Effective calcium carbonate is the calcium carbonate used to bond adjacent soil particles [55], and a smooth surface is more conducive to the production of effective calcium carbonate, increasing the existing bond between particles [56]. The peak stress decreases as the size of the rubber particles increases. This may be due to the gradual transformation of rubber particles from filling pores to becoming part of the soil skeleton, resulting in a weakening of the skeleton’s carrying capacity and the appearance of relatively large pores. In a comparison of Figure 8a–c, it is apparent that shear strength increases with increasing perimeter pressure. Under high-peripheral-pressure conditions, the contact between the particles is tighter, leading to an increase in the friction coefficient between the particles [57]. Specimens with different rubber particle contents had higher peak stresses (Figure 8c) under high-perimeter-pressure conditions compared to low-perimeter-pressure ones (Figure 8a), suggesting that the effect of rubber particle content is related to external loading.

### 3.2. CPO-CNN-LSTM Model Prediction Results

**Figure 9:**
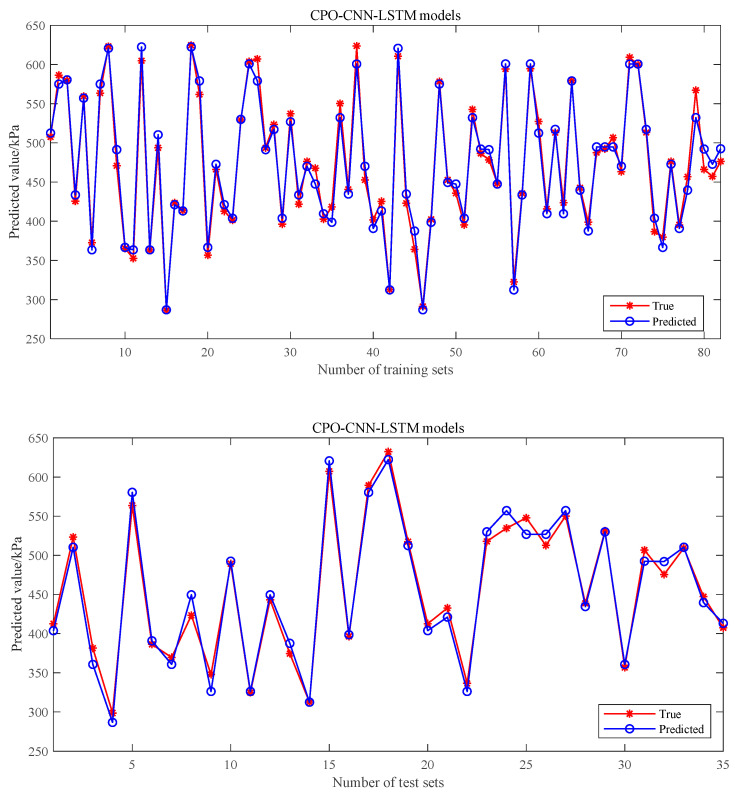
True vs. predicted shear strength for training and test datasets using CPO-CNN-LSTM models.

Figure 9 demonstrates the results of the prediction of shear strength using the CPO-CNN-LSTM method in comparison with the measured shear strength properties analyzed. The coefficient of determination (*R*^2^) is used to assess the goodness of fit of the machine learning model for predicting the sample shear strength, and it takes a value ranging from 0 to 1, where 1 indicates that the predicted values exactly match the actual values. The *R*^2^ values of the shear strength prediction of the CPO-CNN-LSTM model for the training dataset and the test dataset are 0.98 and 0.97, respectively, which indicate that the model explained 98% and 97% of the variance in the shear strength values. This result confirms that the CPO-CNN-LSTM model is able to effectively capture the complex relationship between input features and shear strength, demonstrating excellent prediction performance. Although the *R*^2^ value of the test dataset is slightly lower than that of the training dataset, this small difference is within the expected range, as the model may not be able to fully generalize in the face of unseen data. Nonetheless, the high *R*^2^ values on the test dataset still indicate that the CPO-CNN-LSTM model possesses strong predictive capabilities, is able to efficiently learn potential patterns in the data, and exhibits good generalization capabilities in unseen instances.

### 3.3. Comparative Discussions

In order to validate the effectiveness of the proposed method, this study employs a variety of algorithms for comparative analysis. Specifically, the compared algorithms include convolutional neural network (CNN), long short-term memory (LTSM) network, Extreme Learning Machine (ELM), Random Forest (RF), Support Vector Machine (SVM), and Radial Basis Function (RBF) algorithms. The significant superiority of the CPO-CNN-LSTM algorithm in terms of performance is further verified by a comprehensive comparison of the prediction results of different algorithms.

**Figure 10:**
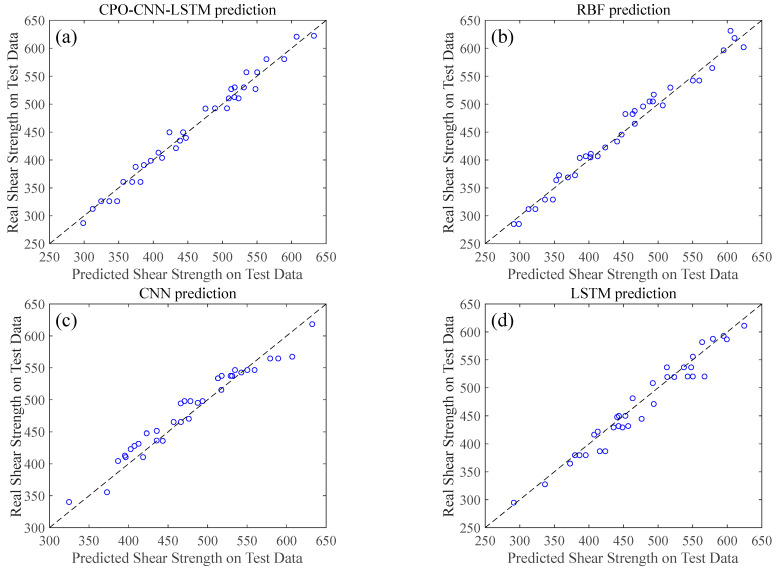

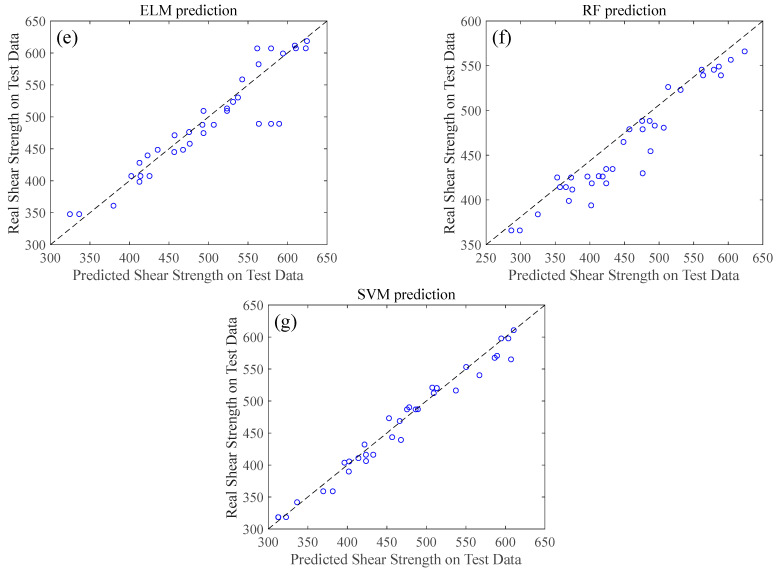
Prediction results of different algorithmic models: (**a**) CPO-CNN-LSTM model, (**b**) RBF model, (**c**) CNN model, (**d**) LSTM model, (**e**) ELM model, (**f**) RF model, and (**g**) SVM model.

Table 4 demonstrates the *R*^2^ values of the different algorithmic models. In the results of the training dataset, it can be seen that the *R*^2^ values of all the models are distributed between 0.92 and 0.98, indicating that these models have a high degree of fit to the data, with the CPO-CNN-LSTM model having the most outstanding goodness of fit. In the test dataset, all the models performed moderately well in predicting the shear strength of EICP-solidified rubber–clay mixtures, but the predictive performance of the RF model was relatively weak. Figure 10 illustrates the comparison between the predicted and actual values of shear strength for different algorithmic models in the test set, where the dots on the straight line in the middle indicate that the predicted values are in perfect agreement with the true values. The results in the figure further confirm the effectiveness of all models in predicting the shear strength of EICP-solidified rubber–clay mixtures. The prediction bias of the RF algorithm is more obvious, while the prediction performance of the RBF model is better than that of RF, but still not as accurate as that of the CPO-CNN-LSTM model. It is shown that the improved machine learning algorithm can significantly improve the accuracy of shear strength prediction, but its effectiveness is affected by a number of factors: (1) the quality of the dataset, which affects the performance of the model due to the fact that the same soil samples may produce variable bias under different experimental conditions; (2) the size of the dataset, given that a limited amount of data may limit the modeling ability, and a larger dataset combined with an adaptive sampling approach helps to improve the regression effect of the model; (3) the choice of independent variables, as different combinations of variables may significantly affect the complexity and predictive ability of the model [35].

## 4. Conclusions

This study examined the potential of employing EICP as a consolidation technique with rubber particles (2.5% to 10% by dry mass) to enhance the shear strength of weak clays. Three different sizes of rubber particles, designated as Rubber A (fine), Rubber B (medium), and Rubber C (coarse), were investigated. The CPO-CNN-LSTM model was developed to predict the experimental outcomes and compared with other commonly used machine learning methods. The main conclusions are as follows:

(1) The addition of rubber particles significantly improves the shear strength of EICP-treated clays, with optimal enhancement observed at a 5% rubber particle content for all sizes. Moreover, smaller rubber particles exhibited superior improvement in shear strength for any given content.
(2) In this study, a CPO-CNN-LSTM model was constructed by integrating CNN and LSTM algorithms and optimizing them using the CPO algorithm. The *R*^2^ values for the training and test datasets reached 0.98 and 0.97, respectively. Comparative analysis revealed that the CPO-CNN-LSTM model outperformed other commonly used models in predicting the shear strength of EICP-solidified rubber–clay mixtures.
(3) We recognize that there may be some limitations in the experimental process, such as the relatively limited amount of data and the control of experimental conditions. In addition, certain assumptions and simplifications of the model may have some impact on the accuracy of the results. Based on the results and shortcomings of this study, we plan to carry out the following work in the future: first, to further expand the experimental dataset size to improve the reliability and generalization ability of the model; second, to explore more types of input variables and model structures to further improve prediction accuracy; and third, to apply the methodology of this study to other related fields to verify its generalizability and effectiveness.

## Figures and Tables

**Table 1:** Index properties of the soil.

Liquid Limit (%)	Plastic Limit (%)	Plasticity Index (%)	Specific Gravity
42.80	24.06	18.74	2.69

**Table 2:** XRF testing results for the soil.

Component	SiO_2_	Al_2_O_3_	CaO	Fe_2_O_3_	MgO	K_2_O	TiO_2_	Na_2_O	P_2_O_5_	SrO	Rb_2_O
Mass (%)	52.96	18.75	11.80	7.36	3.86	3.42	0.79	0.40	0.21	0.05	0.01

**Table 3:** Engineering properties of rubber particles.

Property	Rubber A	Rubber B	Rubber C
Density (g/cm^3^)	1.09	1.11	1.13
Elastic modulus (MPa)	11.97	16.72	22.96
Softening temperature (°C)	175	175	175

**Table 4:** *R*^2^ of different algorithmic models.

*R* ^2^	CPO-CNN-LSTM	RBF	CNN	LSTM	ELM	RF	SVM
Training	0.98	0.97	0.96	0.96	0.95	0.92	0.97
Testing	0.97	0.96	0.94	0.94	0.85	0.82	0.96

## Data Availability

The data presented in this article are available within the article.
